# Lethality of *Brucella microti* in a murine model of infection depends on the *wbkE* gene involved in O-polysaccharide synthesis

**DOI:** 10.1080/21505594.2019.1682762

**Published:** 2019-11-02

**Authors:** Safia Ouahrani-Bettache, María P. Jiménez De Bagüés, Jorge De La Garza, Luca Freddi, Juan P. Bueso, Sébastien Lyonnais, Sascha Al Dahouk, Daniela De Biase, Stephan Köhler, Alessandra Occhialini

**Affiliations:** a IRIM, CNRS, University Montpellier, INSERM, Montpellier, France; b Unidad de Tecnología en Producción y Sanidad Animal, Centro de Investigación y Tecnología Agroalimentaria, Instituto Agroalimentario de Aragón, Universidad de Zaragoza, Zaragoza, Spain; c Laboratorio Agroalimentario, Gobierno de Aragón, Zaragoza, Spain; d CEMIPAI, CNRS, University Montpellier, Montpellier, France; e Department of Biological Safety, German Federal Institute for Risk Assessment, Berlin, Germany; f Department of Medico-Surgical Sciences and Biotechnologies, Sapienza University of Rome, Laboratory affiliated to the Istituto Pasteur Italia – Fondazione Cenci Bolognetti, Latina, Italy

**Keywords:** *Brucella*, virulence, lipopolysaccharide (LPS), O-polysaccharide, glycosyltransferase, rough phenotype, atomic force microscopy

## Abstract

*Brucella microti* was isolated a decade ago from wildlife and soil in Europe. Compared to the classical *Brucella* species, it exhibits atypical virulence properties such as increased growth in human and murine macrophages and lethality in experimentally infected mice. A spontaneous rough (R) mutant strain, derived from the smooth reference strain CCM4915^T^, showed increased macrophage colonization and was non-lethal in murine infections. Whole-genome sequencing and construction of an isogenic mutant of *B. microti* and *Brucella suis* 1330 revealed that the R-phenotype was due to a deletion in a single gene, namely *wbkE* (BMI_I539), encoding a putative glycosyltransferase involved in lipopolysaccharide (LPS) O-polysaccharide biosynthesis. Complementation of the R-strains with the *wbkE* gene restored the smooth phenotype and the ability of *B. microti* to kill infected mice. LPS with an intact O-polysaccharide is therefore essential for lethal *B. microti* infections in the murine model, demonstrating its importance in pathogenesis.

## Introduction

Brucellae are Gram-negative facultative intracellular coccobacilli causing brucellosis, a major bacterial zoonosis with 500,000 human cases globally reported every year [1]. In the last decade, new species of *Brucella*, such as *Brucella microti, Brucella inopinata* and isolates from Australian rodents and amphibians, have been described [2]. These strains are metabolically more active, acid-resistant and fast-growing when compared to the well-known classical, human-pathogenic *Brucella* species, which include *Brucella abortus, Brucella melitensis, Brucella suis* and *Brucella canis* [2–7]. Their isolation from hitherto unknown wildlife hosts and the environment raised the question whether *Brucella* may be transmitted from these reservoirs to livestock and humans living in officially brucellosis-free areas of the world.

*B. microti* was isolated from common vole, red fox, wild boar, and soil in Central Europe and, more recently, from a domestic marsh frog farm [8,9]. Phylogenetically, this species is closer to those pathogenic for human and livestock than to the group of newly described atypical species/strains [2,10]. However, in the absence of clinical reports, the pathogenic potential of *B. microti* remains to be verified. We were the first to describe that, unlike the classical *Brucella* species, *B. microti* is lethal in mice when injected intraperitoneally (i.p.) at a standard dose [11]. The lethal phenotype in mice depends on the type IV secretion system VirB [12] and is also a general unambiguous criterion to establish if a specific *Brucella* gene plays a role in virulence of *B. microti*, as wild-type bacteria kill the murine host at the infection dose of 10^5^ CFU (colony-forming units); in contrast, in classical species virulence has been correlated to the capacity of a strain to establish or maintain various degrees of chronic infection of the spleen and/or the liver, necessitating repeated bacterial enumeration in these organs to follow up the course of infection. On the other hand, at sub-lethal doses (≤10^4^ CFU), *B. microti* is rapidly cleared from infected mice, never gives rise to chronic infection and confers protection [11]. Lethality in mice was later also demonstrated for *B. inopinata* BO1 and *Brucella* strain 83–210 [13]. We and others assumed that the ability of these *Brucella* species to kill the murine host may be due to differences in surface antigens, in particular the structure of lipopolysaccharide (LPS) with a possibly higher endotoxic potential [13,14]. Because of its low endotoxicity, the LPS of classical *Brucella* species is considered as non-canonical in comparison with that of *Escherichia coli* and other pathogenic bacteria, enabling *Brucella* to establish chronic infections and evade TLR4 detection [15–17]. The LPS is a major component of the outer membrane and consists of three key elements: (1) the lipid A, which provides the hydrophobic LPS anchor in the outer membrane, (2) an inner and outer core composed of branched-chain oligosaccharides, and (3) an O-polysaccharide (O-PS), linked to the outer core and protruding into the extracellular environment. In *Brucella*, the O-PS is characterized by a homopolymeric linear chain of N-formyl-perosamine residues linked via α-1,2 and/or α-1,3 glycosidic bonds [18]. Depending on the relative abundance and distribution of these bonds, the O-PS provides the A, M, and common (C) epitopes widely used for serotyping [19,20]. Depending on the presence or absence of the O-PS, the colony phenotype is either smooth (S) or rough (R). All *Brucella* species that infect humans and livestock are naturally S, except for *Brucella ovis* and *B. canis* [21]. For vaccination of livestock against brucellosis, S- and R-strains have been used [22].

Notably, a specific interaction between intact LPS and the lipid rafts in phagocytic cells is responsible for the selective entry of *Brucella* S-strains into the host cells and trafficking along the endocytic pathway [23–26]. In contrast, R strains do not enter the cell through the lipid rafts and are rapidly eliminated [25].

In this study, we characterized a spontaneous R-mutant (*Bm*R^SM^) of the *B. microti* reference strain CCM4915^T^. Its complete genome sequence helped to identify a mutation inactivating the *wbkE* gene, known to be involved in the synthesis of O-PS [27]. To correlate this mutation with the R phenotype and virulence, we constructed a knock-out mutant (*Bm*R^Δ*wbkE*^) by allelic exchange. The fate of R^SM^, R^Δ*wbkE*^ and their complemented strains in cellular and murine infection models was studied and compared to that of the zoonotic strain B. suis 1330.

## Material and methods

### Bacterial strains, culture conditions and phenotypic characterization

*E. coli* and *Brucella* strains (Table 1) were grown under aerobic conditions at 37°C in Luria Bertani (LB, Invitrogen) and Tryptic Soy (TS, Difco) media, respectively. When necessary, media were supplemented with kanamycin or ampicillin at 50 µg/ml, or with chloramphenicol at 25 µg/ml. All experiments with viable *Brucella* were performed in a BSL-3 facility. The smooth (S) and rough (R) phenotypes of *Brucella* were assessed by crystal violet staining [28] and by agglutination tests using anti-R polyclonal antiserum and anti-A and anti-M monospecific sera (ANSES, France). Bacterial morphology was observed by atomic force microscopy (AFM).

**Table 1.** Bacterial strains, plasmids, and primers used in this study.

Bacterial strains	Acronyms	Description	Reference
*E. coli* DH5α	*E. coli*	supE44 Δl*acU169*(ϕ80*lacZ*ΔM15)*hsdR17recA1endA1 gyrA96thi-1 relA1*λpir	Invitrogen
*B. microti* CCM4915^T^	*Bm*S^WT^	Wild-type reference strain, smooth phenotype	[8]
*B. microti* R^SM^	*Bm*R^SM^	Spontaneous mutant of *Bm*S^WT^, rough phenotype	This work
*B. microti* R^SM^ +pBBR-*wbkE*	*Bm*R^SM^p*^wbkE^*	Complemented strain of *Bm*R^SM^ carrying the *wbkE* gene in plasmid pBBR1MCS	This work
*B. microti* Δ*wbkE*	*Bm*R^Δ*wbkE*^	Deletion mutant of *Bm*S^WT^ in which the *wbkE* gene is replaced by a kanamycin cassette	This work
*B. microti* Δ*wbkE* +pBBR-*wbkE*	*Bm*R^Δ*wbkE*^p*^wbkE^*	Complemented strain of *Bm*R^Δ*wbkE*^ carrying the *wbkE* gene in plasmid pBBR1MCS	This work
*B. suis* 1330	*Bs*S^WT^	Wild-type reference strain, smooth phenotype	ATCC 23444
*B. suis* Δ*wbkE*	*Bs*R^Δ*wbkE*^	Deletion mutant of *Bs*S^WT^ in which the *wbkE* gene is replaced by a kanamycin cassette	This work
*B. suis* Δ*wbkE* +pBBR-*wbkE*	*Bs*R^Δ*wbkE*^p*^wbkE^*	Complemented strain of *Bs*R^Δ*wbkE*^ carrying the *wbkE* gene in plasmid pBBR1MCS	This work
**Plasmids**			
pGEM®-T		T/A cloning vector with ampicillin resistance marker	Promega
pUC4K		Plasmid vector carrying a kanamycin resistance cassette (KanR)	GE Healthcare
pBBR1MCS		*E. coli*/*Brucella* shuttle vector with chloramphenicol resistance marker	[29]
pGEM-T-AB		pGEM-T carrying the AB PCR-fragment with sequences up- and downstream of *Brucella wbkE*	This work
pGEMT-AB-Kan		pGEM-T-AB carrying KanR in *Eco*RI site of the AB fragment	This work
pBBR1MCS*-wbk*E		pBBR1MCS carrying the *wbkE* PCR-fragment including the native gene with 398 bp up- and 600 bp downstream regions cloned into *Xho*I-*Sac*I-sites	This work
Primers	Fragment	Sequence (5ʹ- 3ʹ)^1^	Size (base pairs)
A-BMI_I539-For	A	GCAGTGGATCGTGGTGTATG	526 bp
A-BMI_I539 *Eco*RI-Rev		TGAGGTTTCATAGGCCCATCGAATTCCATGAATGGTTCGCTCAATG	
B-BMI_I539-*Eco*RI-For	B	CATTGAGCGAACCATTCATGGAATTCGATGGGCCTATGAAACCTCA	595 bp
B-BMI_I539-Rev		ACATTAATCGCCCGACACTC	
BMI_I539-*Xho*I-For	*wbkE*	**GCGCCTCGAG**AGTTGCCATCATGAGCTTGT	1928 bp
BMI_I539-*Sac*I-Rev		**GCGCGAGCTC**TATCGGAAACAGTCGTGGTC	

^1^Restriction sites are underlined, and non-homologous regions are indicated by bold type. Size of the fused AB PCR-fragment obtained using both primers A-BMI_I539-For and B-BMI_I539-Rev, was 1075 bp. All PCRs were performed with Pfx DNA polymerase (Invitrogen) using *B. microti* genomic DNA as matrix.

### DNA analysis and mutant strains construction

Genomic DNA of *B. microti* was isolated using the Qiagen Mini Kit. Whole-genome shotgun sequencing of the spontaneous rough strain (*Bm*R^SM^) was performed using Illumina paired-end sequencing with a library insert size of 300 bp and an average target coverage of 484 x (GATC). *Bm*R^Δ*wbkE*^ of *B. microti* was obtained by replacing an internal portion of gene BMI_I539 with a Kan^R^ cassette. A recombinant *wbkE*-Kan^R^-containing plasmid derived from pGEM®-T was created as previously described [5,30]. Briefly, two DNA fragments (A, 526 bp and B, 595 bp) each carrying an *Eco*RI restriction site in a 46-bp homology region at the 3ʹ- and 5ʹ-end, respectively, were amplified by PCR. The fragments were fused in a second PCR, to yield fragment AB (1075 bp) containing the *Eco*RI site in the middle and missing 608 out of 1110 bp of the target gene BMI_I539 (i.e. from positions 71 to 678; Table 1). Following cloning of fragment AB in pGEM-T, the resulting plasmid (pGEM-T-AB; Table 1) was digested with *Eco*RI and ligated with the Kan^R^ cassette (1282 bp) excised from plasmid pUC4K. The resulting plasmid (pGEM-T-ABKan, 5357 bp; Table 1) was electroporated into *B. microti*. The Kan^R^/Amp^S^ clones arising from double-crossover were selected and verified by PCR (primers in Table 1). Same constructions and protocols were used to obtain the Δ*wbkE* mutant of *B. suis* (*Bs*R^Δ*wbkE*^; Table 1).

Using electroporation, the three mutant strains (*Bm*R^SM^, *Bm*R^Δ*wbkE*^, *Bs*R^Δ*wbkE*^) were complemented with pBBR1MCS-*wbkE* vector, containing the wild-type *B. microti wbkE* gene and its up- and downstream regions (Table 1).

### Atomic force microscopy

Bacteria were grown to stationary phase in TS, washed in PBS, fixed for 1 h in 2.5% glutaraldehyde and stored in PBS at 5 × 10^9^ bacteria/ml. FluoroDish™ cell culture dishes (World Precision Instruments, UK) were coated overnight at 4°C with 0.1% poly-L-lysine, washed with PBS, air dried and stored at 4°C. Bacteria were diluted 20-fold in PBS and added to the functionalized dish. Images were recorded with qp-BioAC CB2 cantilevers using the quantitative imaging mode available on the NanoWizard IV AFM (JPK Instruments – Bruker). The applied force was kept at 0.3 nN, and a constant approach/retract speed of 80 µm/s (z range of 800 nm).

### Macrophage infection with Brucella strains

Murine J774A.1 macrophage-like cells were infected with *B. microti* and *B. suis* strains at a multiplicity of infection (MOI) of 20 as described previously [11]. All experiments were performed in triplicate.

### Infection of Balb/c mice with B. microti strains

Approved animal experimentation guidelines were followed in the mouse experiments and the working protocol was approved by the CITA ethical animal experiment committee. A procedure for the assessment of pain, distress and discomfort in experimental animals, adapted from [31] was followed, assigning a score to each animal regarding several variables (weight loss, appearance, spontaneous behavior, responses to external stimuli and clinical signs). If the score rose to 15–20 points prior to spontaneous death, animals were euthanized and considered as having succumbed to infection. Bacteria were inoculated i.p [11]. To test the lethality of the bacterial strains, groups of six 9-weeks-old Balb/c female mice (Janvier Labs) each were infected i.p. with 10^5^ CFU of the wild-type, *Bm*R^Δ*wbkE*^ and complemented *Bm*R^Δ*wbkE*^ strains or with 10^8^ and 10^9^ CFU of *Bm*R^SM^ and *Bm*R^Δ*wbkE*^ mutant strains. Mice survival was monitored over a period of 25 days post-infection (d.p.i.).

To study the course of infection in mice, Balb/c were inoculated i.p. with a dose of 10^4^ CFU of *B. microti* strains. Five mice per strain were sacrificed at 3, 14 and 21 d.p.i. Following mice euthanasia by CO_2_ asphyxiation, spleens were aseptically collected, weighed, homogenized, serially diluted and plated onto TS agar for viable counts of *Brucella*. The significance of differences between strains was analyzed by the Student t-test. P values ≤ 0.05 were considered significant.

### Sequence accession number

The genomic DNA sequence of *Bm*R^SM^ has been deposited in the SRA database (NCBI) under the accession number PRJNA545613.

## Results

### *A spontaneous rough mutant of* B. microti *shows increased colonization of macrophages and is avirulent in mice*

Following the first plating of *B. microti* CCM4915^T^ on TS agar and staining with crystal violet, a rough colony was observed. The colony was picked, subcultured three times and stained again with crystal violet: the rough phenotype remained stable for all colonies on plate. This strain was named *B. microti* rough spontaneous mutant and abbreviated *Bm*R^SM^.

**Figure 1.**
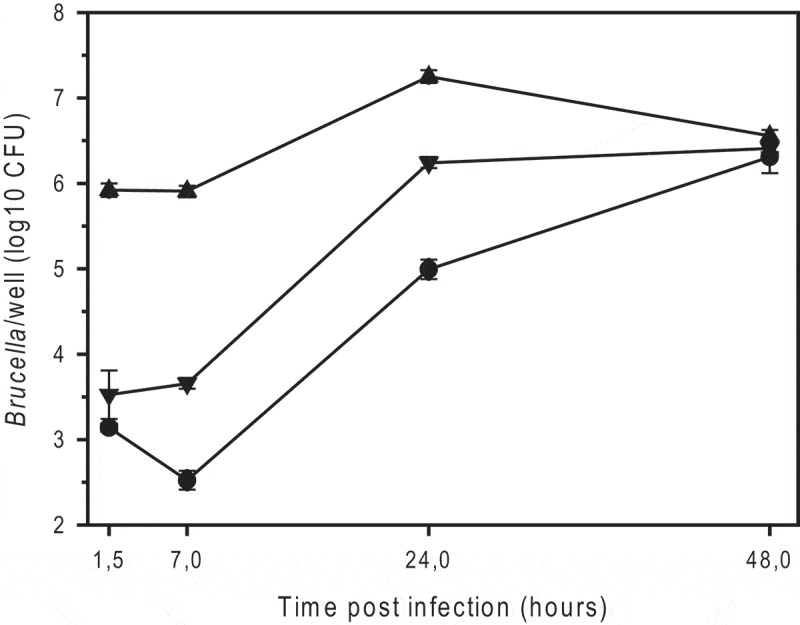
Intracellular replication of smooth *B. microti* CCM4915^T^ (*triangle down*), the spontaneous rough mutant of *B. microti* (*Bm*R^SM^; *triangle up*), and smooth *B. suis* 1330 (*circle*), in murine J774A.1 macrophage-like cells. The number of colony forming units (CFU) was determined by plating serial dilutions on TS agar plates after 2 or 3 days of incubation at 37°C for *B. microti* and *B. suis*, respectively. The experiments were performed three times in triplicate each. Data are presented as mean values ± SD of one experiment (in triplicate).

It has been reported that rough mutant strains of *B. suis* and *B. melitensis* exhibit reduced intracellular survival in infected macrophages, though entry is improved [28]. *Bm*R^SM^ indeed entered murine J774A.1 macrophage cells approximately 100-fold better than the wild-type strain and *B. suis* 1330, which was used as standard reference (Figure 1). In contrast to *B. suis* and *B. melitensis* rough strains [28], *Bm*R^SM^ replicated 20-30-fold, at least up to 24 hours post-infection (Figure 1).

To characterize the behavior of *Bm*R^SM^ *in vivo*, Balb/c mice were injected i.p. with the sublethal dose of 10^4^ bacteria, as previously published by the authors for the *B. microti* wild-type strain [11]. The number of bacteria recovered from spleen and liver 3 days after inoculation, corresponding to the peak of infection for *B. microti* [11], was reduced by 3.5 and 2.2 logs (P < 0.001), respectively, compared to those previously obtained with the wild-type strain (Table 2) and also confirmed in this work (see later section on “acute murine infection”). Therefore, the rough mutant colonized these organs significantly less than the wild-type, resulting in the lack of a transient acute phase of infection.

**Table 2.** Balb/c mice liver and spleen colonization by *B. microti* S and R^SM^ strains 3 days post-inoculation.

Strains	Bacteria/spleen (log10 CFU)	Bacteria/liver (log10 CFU)
*B. microti* CCM4915^T^ Smooth	6.52 ± 0.16^a^	5.43 ± 0.21^a^
*B. microti* R^SM^	3.09 ± 0.59	3.27 ± 0.31

Results represent means ± SD. Differences between both strains are significant in both organs (P < 0.001).

^a^ Previously published data [11]

### BmR^SM^ strain is characterized by a mutation of the glycosyltransferase-encoding gene wbkE

To identify the mutation(s) responsible for the rough phenotype in *Bm*R^SM^, its genome was sequenced and compared with that of *B. microti* CCM4915^T^, accessible in the NCBI database. Out of 48 variants, 28 were assigned to SNV (Single Nucleotide Variants) and 20 to InDel (Insertion/Deletion) variants. Scrutiny of the variants resulted in retaining of 3 SNV and 4 InDels, fulfilling all the following criteria: (1) located within open reading frames or in the immediate upstream vicinity, (2) causing amino acids substitutions, frameshift- or stop-mutations in the corresponding genes, (3) not located in pseudogenes, and (4) representing the most prevalent variant according to the number of sequencing reads (≥ 90%) with respect to the reference sequence (Supplementary Table S1). Only four mutations were intragenic and affected the following genes: BMI_I525 (2 SNVs), BMI_I539 (1 InDel) and BMI_I1103 (1 SNV), encoding a transposase (ISBm1), a glycosyltransferase (*wbkE*) and a queuine tRNA-ribosyltransferase (*tgt*), respectively. The mutation found in *wbkE* was regarded as the most plausible cause for the rough phenotype, because the homologous gene BMEI1393 of *B. melitensis*, located in a highly conserved cluster of the major (*wbk*) genetic region of LPS synthesis, participates in O-PS biosynthesis [2,27]. Protein sequences of BMI_I539 and BMEI1393 are identical for 368 out of 369 amino acids. In *Bm*R^SM^, deletion of a T at position 452 of *wbkE* causes a frameshift and the generation of a premature stop codon at position 622. The resulting protein sequence is therefore expected to be truncated at position 207.

### Deletion of the B. microti wbkE gene confirms its role in smooth (S-)LPS biosynthesis and results in enhanced macrophage uptake

**Figure 2.**
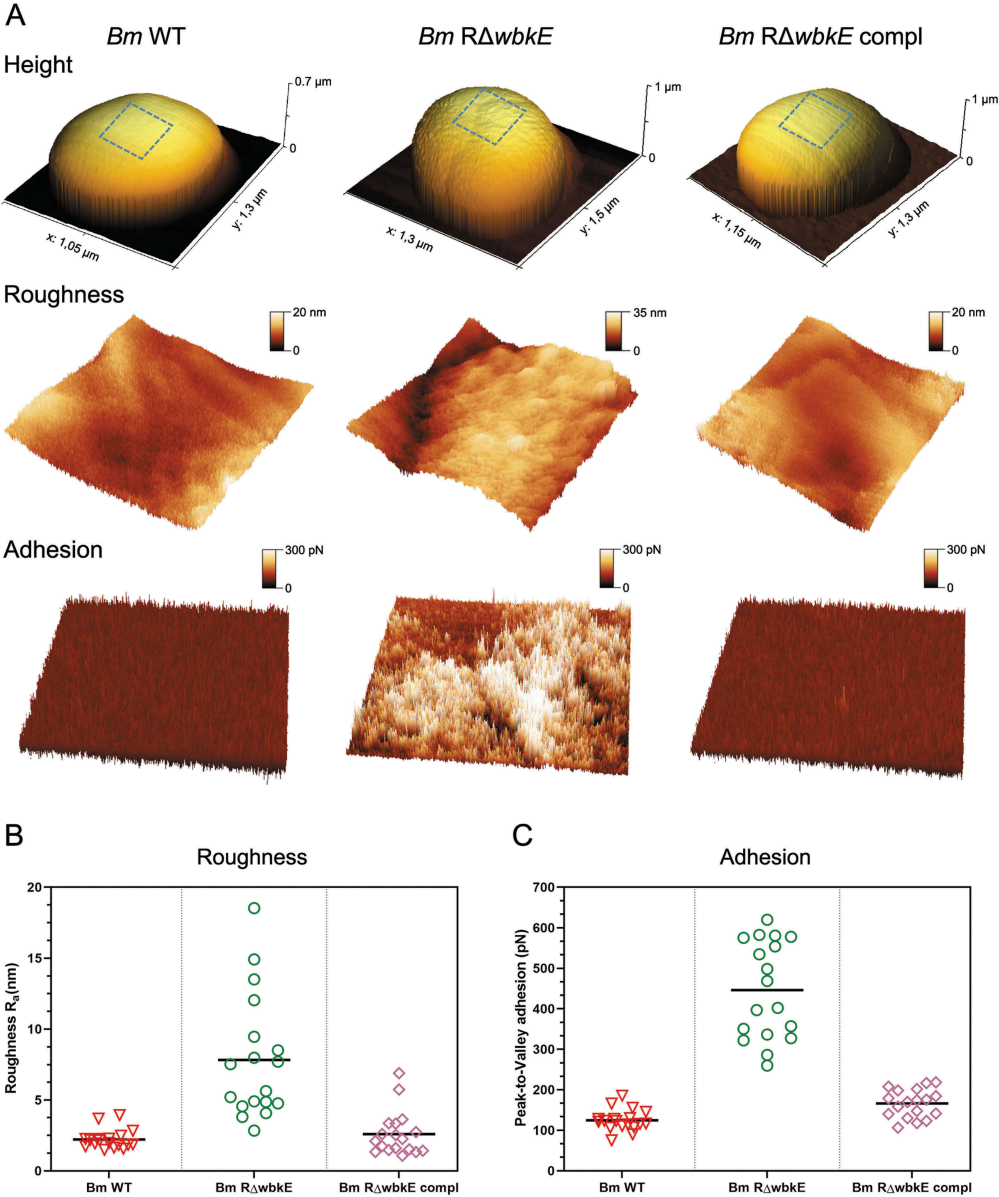
Atomic Force Microscopy (AFM) images of *B. microti* wild-type (*Bm* WT), Δ*wbkE* mutant (*Bm* RΔ*wbkE*) and the complemented Δ*wbkE* mutant (*Bm* RΔ*wbkE* compl). (a) Each column shows from top to bottom the vertical deflection image (height) of the whole bacteria and 0.3 × 0.3 µm^2^ areas of the cell surface, representing roughness and adhesion recorded on the shown bacteria (blue square). Quantitative roughness (b) and adhesion (c) measurements of *Bm* WT, *Bm* RΔ*wbkE* and complemented *Bm* RΔ*wbkE*: 0.5 × 0.5 µm^2^ images were recorded and used for measurements of 0.25 × 0.25 µm^2^ areas to quantify arithmetic roughness R_a_ and adhesion (Peak-to-Valley). n = 9 bacteria/strain. Statistical differences were analyzed by t-test and yielded P values < 0.001 when comparing *Bm* WT or *Bm* R^Δ*wbkE*^ compl with *Bm* R^Δ*wbkE*^. Image analysis was done with Gwyddion [34].

To confirm that the absence of a functional *wbkE* is responsible for the rough phenotype and the reduced virulence of *Bm*R^SM^, we constructed a mutant (*Bm*R^Δ*wbkE*^) by replacing a 608-bp internal fragment of *wbkE* with a Kan^R^ cassette in the parental strain. Colony staining with crystal violet and agglutination with anti-R antiserum [32] confirmed that *Bm*R^Δ*wbkE*^ was rough as *Bm*R^SM^ (Table 3). In parallel, we performed a surface analysis of smooth and rough bacterial strains by atomic force microscopy (AFM). AFM has been established as a powerful imaging technique and allows characterization of the surface morphology of microbial cells at the nanoscale [33]. We used a force-curve-based imaging mode where the AFM tip is pushed toward an area of the cell surface and retracted from it, generating a force vs. separation distance curve encoding information about height, adhesion or elasticity for each image pixel. Surface topography of *B. microti* wild-type and *Bm*R^ΔwbkE^ revealed a uniform, smooth structure for *B. microti* wild-type, in contrast to a jagged, irregular structure for the *Bm*R^ΔwbkE^ mutant, with significantly increased roughness (Figure 2(a,b); Table 3). Mapping of the adhesion forces between the tip and the bacterial surface also revealed the presence of large patches of increased adhesion at the surface of *Bm*R^ΔwbkE^ when compared with the surface of wild-type bacteria, suggesting important differences in the molecular structure of the *Bm*R^ΔwbkE^ mutant surface (Figure 2(a,c); Table 3).

**Table 3.** Phenotypes of S and R strains of *B. microti* and *B. suis.*

			Atomic Force Microscopy	
Strains	Crystal violet staining^a^	Serum agglutination^b^	Roughness, nm ± SD	Adhesion, pN ± SD
*B. microti* CCM4915^T^ Smooth	-	M	2.2 ± 0.67	124.6 ± 26
*B. microti* R^SM^	+	R	ND^c^	ND
*B. microti* R^SM^ + pBBR*-wbk*E	-	M	ND	ND
*B. microti* Δ*wbk*E	+	R	7.8 ± 4.35	446 ± 120.4
*B. microti* Δ*wbk*E + pBBR*-wbk*E	-	M	2.6 ± 1.57	166.1 ± 34.9
*B. suis* 1330 Smooth	-	A	ND	ND
*B. suis* 1330 Δ*wbk*E	+	R	ND	ND
*B. suis* 1330 Δ*wbk*E + pBBR*-wbk*E	-	A	ND	ND

^a^ +, uptake; -, no uptake of crystal violet by the colonies

^b^ with monospecific A, M, or R polyclonal antisera

^c^ not determined

Both rough mutants *Bm*R^Δ*wbkE*^ and *Bm*R^SM^ were then complemented with an intact copy of *wbkE* cloned in vector pBBR1MCS, which restored the smooth phenotype, as evidenced by lack of crystal violet staining and by the agglutination with anti-M antiserum only [8] (Table 3). In addition, AFM confirmed a smooth surface structure of complemented *Bm*R^Δ*wbkE*^, with roughness and adhesion forces back to wild-type levels (Figure 2, Table 3).

**Figure 3.**
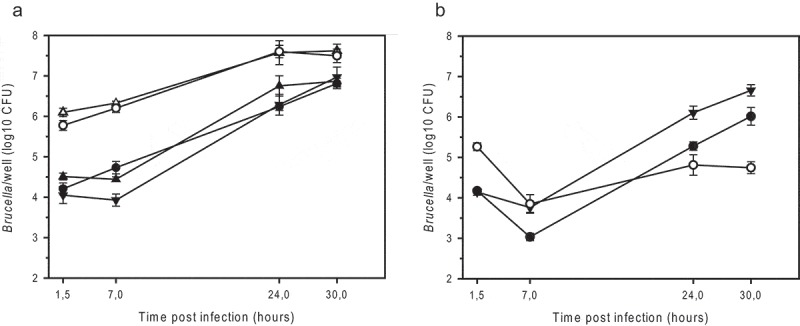
Intracellular replication of smooth and rough strains of *B. microti* (a) and *B. suis* (b) in murine J774A.1 macrophage-like cells. (a) *B. microti* CCM4915^T^ wild-type (*filled triangle down*), the spontaneous R-strain *Bm*R^SM^ (*open triangle up*), the complemented *Bm*R^SM^ mutant (*filled triangle up*), the constructed R-strain *Bm*R^Δ*wbkE*^ (*open circle*), and the complemented *Bm*R^Δ*wbkE*^ mutant (*filled circle*). (b) *B. suis* 1330 wild-type (*filled triangle down*), the constructed R-strain *Bs*R^Δ*wbkE*^ (*open circle*), and the complemented *Bs*R^Δ*wbkE*^ mutant (*filled circle*). The complemented strains expressed native *wbkE* cloned into the replicative plasmid pBBR1MCS. The experiments were performed three times in triplicate each. Data are presented as mean values ± SD of one experiment (in triplicate).

The *Bm*R^Δ*wbkE*^ mutant entered J774A.1 cells to an extent similar to that of the *Bm*R^SM^ mutant (100 times more efficient than the parental strain) and replicated 60-fold over 30 hours (Figure 3(a)). In contrast, *Bm*R^Δ*wbkE*^ and *Bm*R^SM^ strains complemented with an intact copy of *wbkE* infected macrophages like the parental strain and replicated 400- and 250-fold, respectively (Figure 3(a)). An isogenic *wbkE* mutant of *B. suis* 1330 (*Bs*R^Δ*wbkE*^) was also constructed in order to compare its behavior to that of other rough mutants described in the past [25,28]. As expected, the *Bs*R^Δ*wbkE*^ mutant retained crystal violet and agglutinated with anti-R antiserum (Table 3). It colonized the macrophages 12 times more efficiently than the wild-type, but in contrast to R-strains of *B. microti*, a 3-fold reduction in intracellular survival was observed within a period of 30 h, resulting in 100 times lower viable counts when compared to the wild-type (Figure 3(b)). This was very similar to the behavior of the *manB_core_* mutant of *B. suis* 1330 [25]. The complemented *Bs*R^Δ*wbkE*^ strain agglutinated specifically with anti-A antiserum as the wild-type strain (Table 3) and showed wild-type levels of intracellular infection and replication, despite a stronger transitional decrease in the early phase of infection (Figure 3(b)).

### The B. microti wbkE gene is indispensable for acute murine infection

**Figure 4.**
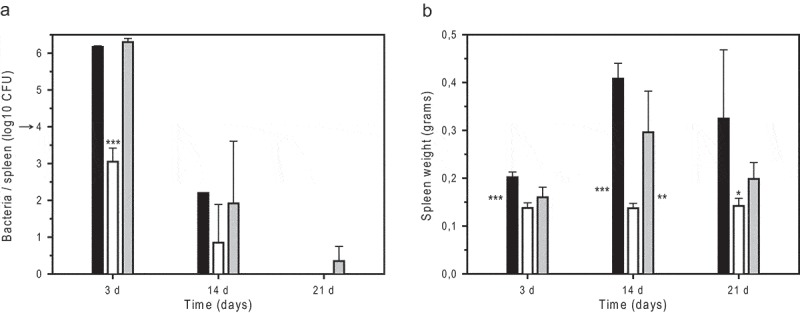
Infection of Balb/c mice with *B. microti* strains: growth and survival of *B. microti* strains in the spleen (a) and spleen weights of infected animals (b) after i.p. inoculation of 10^4^ bacteria. The number of viable *B. microti* CCM4915^T^ wild-type (*black bars*), *Bm*R^Δ*wbkE*^ strain (*open bars*), and complemented *Bm*R^Δ*wbkE*^ mutant (*grey bars*) was determined at days 3, 14, and 21 post-infection. The arrow indicates the infection dose of 10^4^ bacteria. Five mice were sacrificed per bacterial strain and time point, and values represent means ± SD. Asterisks indicate variable significance of the differences between the R-strain and the wild-type (next to left bar) or R-strain and the complemented mutant (next to right bar), or between the R-strain and both the wild-type and the complemented mutant (above middle bar): * P < 0.05; ** P < 0.005; *** P < 0.001.

Infection of Balb/c with 10^4^ CFU of *B. microti* strains showed a 3-logs reduction of *Bm*R^Δ*wbkE*^ in the spleen at day 3 post-injection, as compared to the *wbkE*-complemented mutant and wild-type strains (Figure 4(a)), confirming the incapacity of a Δ*wbkE* mutant strain to establish an acute phase of infection in the host (see also Table 2). The acute infection phase observed with the wild-type and the *wbkE*-complemented strains was followed by an increase of the spleen weight until at least day 14, reflecting an inflammatory response (Figure 4(b)). In contrast, mice infected with *Bm*R^Δ*wbkE*^ did not gain spleen weight. A similar finding was obtained when the strain *Bm*R^SM^ was injected i.p. at 10^4^ CFU, as spleen weights remained unchanged at day 3 and day 14 (0.09 ± 0.007 and 0.10 ± 0.015, respectively; P < 0.001), confirming a reduced immune response induction with the *wbkE*-mutant rough strains.

### The wbkE gene is essential for the lethal character of B. microti infections in Balb/c mice

Our previous work showed that the intra-peritoneal injection of 10^5^ CFU of *B. microti* CCM4915^T^ caused the death of 83% of the Balb/c mice within four days of infection [11]. To investigate the possible involvement of the *wbkE* gene in the lethal outcome of a *B. microti* infection, the susceptibility of Balb/c mice infected with *Bm*R^SM^ or *Bm*R^Δ*wbkE*^ was compared to that observed following infection with the *B. microti* CCM4915^T^ wild-type and the complemented *Bm*R^Δ*wbkE*^ strains, over a monitoring period of 25 days (Table 4): 67% and 83% of the mice infected with the standard dose of 10^5^ CFU of the wild-type or the complemented *Bm*R^Δ*wbkE*^ strain, respectively, died between days 2 and 6 post-inoculation. In striking contrast, all the mice infected with 10^5^ CFU of *Bm*R^Δ*wbkE*^ survived without any symptoms. 100% survival was also observed for both R-mutant strains *Bm*R^SM^ and *Bm*R^Δ*wbkE*^ after the injection of 10^8^ CFU. However, when inoculated with 10^9^ CFU of either R-strain, all mice died between days 2 and 6 post-inoculation. These results are even more notable if combined with our preliminary observation (not shown) that *Bm*R^SM^ in Balb/c mice provided protection against *B. microti* wild-type, *B. abortus, B. melitensis* and *B. suis* 1330.

**Table 4.** Lethality of *B. microti* S and R strains in Balb/c mice

Strains	Infection dose (i.p.)	% Mortality^ab^
***B. microti*****CCM4915^T^ Smooth**	10^5^	67
*B. microti* R^SM^	10^8^	0
	10^9^	100
*B. microti Δwbk*E	10^5^	0
	10^8^	0
	10^9^	100
*B. microti Δwbk*E+ pBBR*-wbk*E	10^5^	83

^a^ Each bacterial strain was inoculated to a group of six 9-weeks-old Balb/c female mice ^b^ Over a 25-days period of monitoring, murine death occurred between days 2 and 6 post-inoculation

## Discussion

The non-canonical LPS of classical brucellae lacks endotoxicity and possesses a particular core structure helping to evade the host’s immune system [35,36]. The phenomenon of dissociation, resulting in the conversion of S- to R-phenotype, has been first described for *Brucella* in 1933 [37]. More recently, *B. abortus, B. melitensis* and *B. suis* R-mutants devoid of O-PS have been studied in cellular and murine models of infection [25,27,28,38,39]. These studies consistently showed the relevance of O-PS for virulence. The reduced virulence of R-mutants has been attributed to (1) high sensitivity to complement-mediated lysis in mice [40,41], (2) lipid raft-independent entry into macrophages resulting in enhanced phagolysosome fusion [25], and (3) lack of intracellular replication due to macrophage activation [28]. Monoclonal antibodies specific for common O-PS epitopes of A- or M-dominant classical strains also recognize LPS of the M-dominant *B. microti* reference strain [14], indicating a conserved structure of O-PS. However, anti-R-LPS monoclonal antibodies do not react with *B. microti* LPS, suggesting structural specificities in the core-lipid A moiety of its LPS [14], which may result in enhanced endotoxic properties and possibly explain the killing ability of *B. microti* in the murine model of infection.

Because of the lack of data available on atypical species mutants affected in O-PS biosynthesis, we investigated the virulence properties of a spontaneous R-mutant of *B. microti*, which was fortuitously isolated. Murine infection experiments showed a loss of lethality of this mutant, and subsequent analysis by whole-genome sequencing allowed to link this ability to the *wbkE* gene, encoding a glycosyltransferase located in the major O-PS biosynthesis region, as previously described for *B. melitensis* [27]. Complementation of R-mutants restored wild-type properties including smooth character, reduced macrophage entry and murine lethality, demonstrating that the *Bm*R^SM^ strain was affected in a single LPS biosynthesis gene. In contrast, spontaneous R-mutants of *B. abortus* and *B. melitensis* isolated from macrophage and murine infections were often simultaneously affected in several loci [42]. Cell surface structure analysis by AFM confirmed the smooth character of the wild-type and complemented *Bm*R^Δ*wbkE*^ strains, whereas a distinct irregular surface was recorded for *Bm*R^Δ*wbkE*^, possibly due to exposure of outer membrane proteins and lipid A/outer core disaccharides in the absence of the O-chain [27]. This exposure may also explain the increased adhesion forces observed during the interaction of the AFM tip with the R-mutant surface. Conversely, in a recent report, AFM analysis of two *B. abortus* strains, a wild-type and its isogenic mutant Δ*gmd* R lacking O-chain, shows similar degrees of roughness [43]. Structural differences in the LPS core-lipid A moieties of *B. microti* and *B. abortus* could be a reason for this divergence.

In the macrophage model of infection, *Bm*R^Δ*wbkE*^ and *Bm*R^SM^ replicated to a number of intracellular bacteria higher than that observed for the wild-type, in part due to the increased rate of entry (100 times). The latter was also described previously for R-mutants of classical species, but with variations in intracellular survival, reaching at best the initial level post-phagocytosis [25,28,44]. Interestingly, this was also confirmed for *Bs*R^ΔwbkE^, leading to the hypothesis that *B. suis* and *B. microti* R-mutants may use distinct intracellular trafficking pathways depending on sites of entry and resulting in specific phagolysosome fusion and escape kinetics. Measurement of NO- and TNF-α-production by infected macrophages yielded only minor differences between *Bs*R^ΔwbkE^ and *Bm*R^ΔwbkE^ strains, indicating that these immune mediators were not involved (not shown).

Intramacrophagic replication and high intracellular loads of bacteria are essential for the lethal phenotype of *B. microti in vivo*, with the major virulence factor VirB playing a crucial role [12]. We assume that massive intracellular replication of the pathogen and increased cell lysis results in high concentrations of circulating bacteria, infecting new cells and leading to septic shock and murine death, possibly due to a modified core-lipid A moiety. However, intramacrophagic replication is not sufficient, as shown for the R-mutant. Lack of murine lethality after infection with R-mutants may indeed be explained by increased sensitivity to complement-mediated lysis, keeping the overall concentrations of bacteria too low to trigger an endotoxic shock and, at a sublethal dose, strongly reducing the load of infected macrophages prior to their settling in the spleen and mitigating the inflammatory reaction. The biological potential of dissociation based on enhanced dissemination of S-bacteria in the presence of R-forms inducing cytotoxicity has been discussed in the context of *B. abortus* and *B. melitensis* infections [42,45], but in the case of rough *B. microti*, final bacterial loads in the macrophage are even higher than with the wild-type, making a cytotoxic effect unlikely. Given the different natural habitats of *B. microti* and the observation that *B. microti* strains isolated from soil were rough [32], we speculate that the R-phenotype either confers an advantage to this species outside the host or appears at higher rates due to the absence of selective pressure to which intracellular smooth organism may be exposed.

In surveillance and control of livestock brucellosis, the distinction between infected and vaccinated animals remains a major challenge, since field strains and the commonly used efficient vaccine strains, *B. abortus* S19 and *B. melitensis* Rev1, both possess a S-LPS, which is the relevant diagnostic antigen recognized by anti-*Brucella* antibodies following infection or vaccination. Therefore, O-PS mutants are interesting vaccine candidates, as they do not elicit an antibody response undistinguishable from that induced during active infection. As a matter of fact, *B. abortus* RB51 has been commercialized as a rough vaccine strain, despite its controversial effectiveness and drawbacks [46]. Based on our preliminary data in mice, we suggest that the spontaneous, well-characterized R-strain of *B. microti* described in this study, or a R-mutant deletion strain devoid of the antibiotic resistance marker, might thus be exploited as a potential DIVA (Differentiating Infected from Vaccinated Animals)-vaccine candidate against animal brucellosis, with the potential advantage to confer a broad-range protection against heterologous *Brucella* species pathogenic for livestock.

In summary, we have described R-mutants of the atypical species *B. microti* defective in *wkbE* expression/activity in cellular and *in vivo* models of infection. Together with VirB, the S-LPS was identified as a virulence factor essential for lethality of *B. microti* in the murine model of infection. Further characterization of its LPS structure, elucidation of intracellular trafficking, and additional *in vivo* studies in livestock animals will show whether *B. microti* R-strains are suitable live vaccines against brucellosis.
