# Supplementary material for: Lethality of Brucella microti in a murine model of infection depends on the wbkE gene involved in O-polysaccharide synthesis
Source: Virulence. 2019 Nov 2;10(1):868–78. doi: 10.1080/21505594.2019.1682762 (PMC6844557; doi:10.1080/21505594.2019.1682762)
Supplement: Supplemental Material [file kvir-10-01-1682762-s001.docx]

**Table S1.** **Delimited Single Nucleotide Variants and InDels (Insertion-Deletions) detected in the spontaneous R mutant (R^SM^) strain derived from *B. microti* reference strain CCM4915^T^**

| **CHROMOSOME^1^** | **POSITION^2^** | **REFERENCE BASE^3^** | **OBSERVED BASE^4^** | **QUALITY SCORE^5^** | **COVERAGE^6^** | **A,C,G,T^7^** | **LOCALIZATION** | **GENE ID^8^** | **GENE/POSITION AFFECTED** | **AMINO ACID CHANGE** | **CODON CHANGE** |
| --- | --- | --- | --- | --- | --- | --- | --- | --- | --- | --- | --- |
| **SNV (Single Nucleotide Variants)** | | | | | | | | | | | |
| NC_013119 | 524873 | C | T | 2486.85 | 129 | 0,0,0,131 | INTRAGENIC | BMI_I525 | ISBm1, transposase orfA | Leu/Phe | **C**TT/**T**TT |
| NC_013119 | 524885 | T | C | 1414.08 | 82 | 1,81,1,0 | INTRAGENIC | BMI_I525 | ISBm1, transposase orfA | Ser/Pro | **T**CC/**C**CC |
| NC_013119 | 1071226 | C | T | 7537.51 | 223 | 0,1,0,222 | INTRAGENIC | BMI_I1103 | BMI_I1103 3'end queuine tRNA-ribosyltransferase (*tgt*) | Lys/Asn | AA**C**/AA**T** |
| **INDELS** | | | | | | | | | | | |
|  |  |  |  |  |  | **ALLELE DEPTH^9^** |  |  |  |  |  |
| NC_013118 | 75894 | GA | G | 21752.7 | 554 | 12,540 | UPSTREAM | NA | 49 bp upstream of BMI_II78 (*hemH*) | NA | NA |
| NC_013119 | 810876 | TA | T | 20020.1 | 520 | 11,501 | UPSTREAM | NA | 166 bp upstream of BMI_I827 (hypothetical protein) | NA | NA |
| NC_013119 | 536349 | CT | C | 18997.3 | 505 | 12,483 | INTRAGENIC | BMI_I539 | Glycosyl transferase (*wbkE)* | **frameshift + STOP** | |
| NC_013119 | 144040 | CA | C | 15684.7 | 524 | 38,478 | UPSTREAM | NA | 11 bp upstream of BMI_I128 (*gyrB*) | NA | NA |

^1^ RefSeq ID of the *B. microti* reference strain CCM4915^T^ chromosome on which the SNV occurs.

^2^ Sequence position of the mutation on the corresponding chromosome.

^3^ The reference base in the genome of the *B. microti* strain CCM4915^T^ at the variant site.

^4^ Alternative base observed in the R^SM^ strain (variant).

^5^ The Phred-scaled probability of OBSERVED BASE is computed based on error models designed by the Broad Institute (Boston, USA). Based on the Phred quality score Q = -10 x log P, a value of 10 indicates a 1 in 10 chance of error, while a 100 indicates a 1 in 10^10^ chance. The higher the value, the more accurate is the variant call.

^6^ The total depth of the reads that passed the internal quality control metrics from all reads present at this site.

^7^All the observed bases at the variant position.

^8^ The gene entry associated with the location of the variant call. If present, gene ID is displayed. If not, "NA" is displayed.

^9^ The allele depth, for the reference (left) and the observed variant (right) sequence, is the count of all reads that included the respective alleles.

The read counts also include the poor mapping quality reads, unlike the COVERAGE counts.
